# Effective macrophage clearance of *Klebsiella pneumoniae* requires the inducible nitric oxide synthase iNOS and is independent of reactive oxygen species generated by NADPH oxidase

**DOI:** 10.64898/2026.05.14.724925

**Published:** 2026-05-18

**Authors:** Alexis E. Wilcox, Catherine J. Andres, Elizabeth H. Madigan, Andrew J. Olive, Caitlyn L. Holmes

**Affiliations:** a Department of Microbiology and Immunology, University of Minnesota Medical School, Minneapolis, Minnesota, USA; b Department of Microbiology, Genetics, and Immunology, College of Osteopathic Medicine, Michigan State University, East Lansing, MI, USA; c Center for Immunology, University of Minnesota Medical School, Minneapolis, Minnesota, USA

## Abstract

*Klebsiella pneumoniae* is a leading cause of pneumonia and bacteremia and is especially dangerous in healthcare settings. Despite massive clinical significance, the mechanisms used by macrophages to kill *K. pneumoniae* are not well defined. Macrophages are critical for controlling *K. pneumoniae* as mice lacking monocyte-derived or alveolar macrophages have higher bacterial tissue burdens and mortality. Two prominent mechanisms used by macrophages to kill bacteria are the production of reactive oxygen species (ROS) via the NADPH oxidase NOX2 and reactive nitrogen species (RNS) via the inducible nitric oxide synthase iNOS. Previously, we found that *K. pneumoniae* uses similar genetic factors to survive during bacteremia and within macrophages. The ability of these factors to enhance intracellular fitness was significantly correlated with resistance against RNS, not ROS. Here, we aimed to define whether macrophage ROS and RNS contribute to intracellular *K. pneumoniae* clearance. Using wild-type, *Cybb*^−/−^, and *Nos2*^−/−^ cells, we measured *K. pneumoniae* survival within macrophages lacking such defenses. NOX2 was dispensable for *K. pneumoniae* clearance, and ROS was undetectable in *K. pneumoniae*-infected macrophages. We confirmed that ROS was undetectable within alveolar-like macrophages, indicating a conserved ROS evasion phenotype across macrophage subsets. Instead, iNOS significantly contributed to macrophage clearance of *K. pneumoniae* and enhanced cytokine production. iNOS likely enhances *K. pneumoniae* clearance through coordination of immunity and RNS. Activation of pathways upstream of iNOS may be the most relevant to supporting effective macrophage control of *K. pneumoniae*. This study defines unexpected differential roles for ROS and RNS in macrophage clearance of *K. pneumoniae*.

## Introduction

Sepsis is immune dysregulation that occurs in response to infection and causes immense global morbidity and mortality (1, 2). Bacterial pneumonia and bacteremia are among the most common initiators of sepsis, accounting for >65% of cases (3). The Gram-negative species *Klebsiella pneumoniae* is a leading cause of both pneumonia and bacteremia (4–7). *K. pneumoniae* is prominent in healthcare settings, where aging and immunocompromised individuals are at higher risk for infection mortality (8, 9). Antibiotic resistance among *K. pneumoniae* strains is increasing, resulting in persistent infections that are difficult to treat (10). In 2024, the World Health Organization named *K. pneumoniae* as the leading bacterial priority pathogen due to this high virulence and antimicrobial resistance (11). Given the clinical relevance of *K. pneumoniae* and its significant impact to global health, it is critical to understand host-pathogen interactions during these infections (9).

*K. pneumoniae* infections are highly inflammatory. In murine pneumonia, inflammatory cytokines, such as TNFα, are significantly elevated in the lungs shortly after *K. pneumoniae* infection (12). Infected lung tissue also demonstrates substantial recruitment of neutrophils and monocyte-derived cells (12, 13). Macrophages are critical for host defense against *K. pneumoniae* as mice lacking monocyte-derived or alveolar macrophages have higher *K. pneumoniae* burden in lung tissue and reduced survival during infection (14–16). Since monocyte-derived macrophages are present across infected tissues, understanding how these cells kill *K. pneumoniae* is necessary for defining pathogen containment across sites even after dissemination has occurred. For alveolar macrophages, defining bacterial evasion mechanisms may illuminate intervention opportunities for preventing the dissemination of disease. Despite the importance of macrophages in controlling infection, mechanisms that support effective clearance of internalized *K. pneumoniae* are minimally described.

Macrophages typically internalize pathogens into phagosomes that fuse with lysosomes (17, 18). Phagolysosome maturation initiates assembly of the phagocyte NADPH oxidase (NOX2) onto its membrane and results in the release of reactive oxygen species (ROS) into the compartment. In parallel, cytosolic inducible nitric oxide synthase (iNOS) generates reactive nitrogen species (RNS) which diffuse passively across the phagolysosome membrane to reach bacteria. Collectively, ROS and RNS are prominent mechanisms used to kill internalized pathogens (19, 20). ROS and RNS differ in their induction pathways and effects on microbes (20). The primary product of NOX2 is superoxide (O_2_^−^), while the primary product of iNOS is nitric oxide (NO^−^). However, these radicals can form other oxygen and nitrogen-based radicals, including hydrogen peroxide (H_2_O_2_) and nitrite (NO_2_^−^). Such agents interact with various bacterial targets. For example, H_2_O_2_ damages DNA while RNS inhibits bacterial respiration (20). Thus, the distinct targets of ROS and RNS indicate that NOX2 and iNOS may have differential contributions to immune stress against *K. pneumoniae,* but this has not been explored in the context of macrophages.

Previously, we used a panel of 53 *K. pneumoniae* mutants with fitness defects during bacteremia and assessed the survival of each after exposure to extracellular ROS and RNS, or the macrophage intracellular environment (21). We discovered that a mutant’s ability to resist macrophage-mediated stress was significantly correlated with the ability to resist nitrosative stress. However, no significant correlation was observed between intracellular fitness and oxidative stress resistance. The extent of *K. pneumoniae* resistance against nitrosative stress was also linked to increased bacteremia fitness, but this trend was not observed for oxidative stress resistance. These data indicate that *K. pneumoniae* resistance to nitrosative stress may be particularly relevant during bacteremia and for intracellular survival. However, our previous work did not investigate whether macrophages specifically require ROS and RNS to eliminate *K. pneumoniae*. As such, the role of these stressors in macrophage defense against *K. pneumoniae* remains unclear.

Here, we aimed to define the contributions of macrophage NOX2 and iNOS to the clearance of intracellular *K. pneumoniae*. We discovered that ROS and NOX2 are dispensable for macrophage clearance of *K. pneumoniae*. Instead, RNS and iNOS significantly enhanced macrophage killing of *K. pneumoniae*. Our data reveal surprising insight into host-pathogen interactions by demonstrating that *K. pneumoniae* blocks the initiation of macrophage ROS. Enhancing pathways that activate RNS production via iNOS may be highly relevant for promoting host control of these dangerous infections.

## Results

### iNOS significantly enhances the killing of intracellular *K. pneumoniae*.

**Figure 1.**
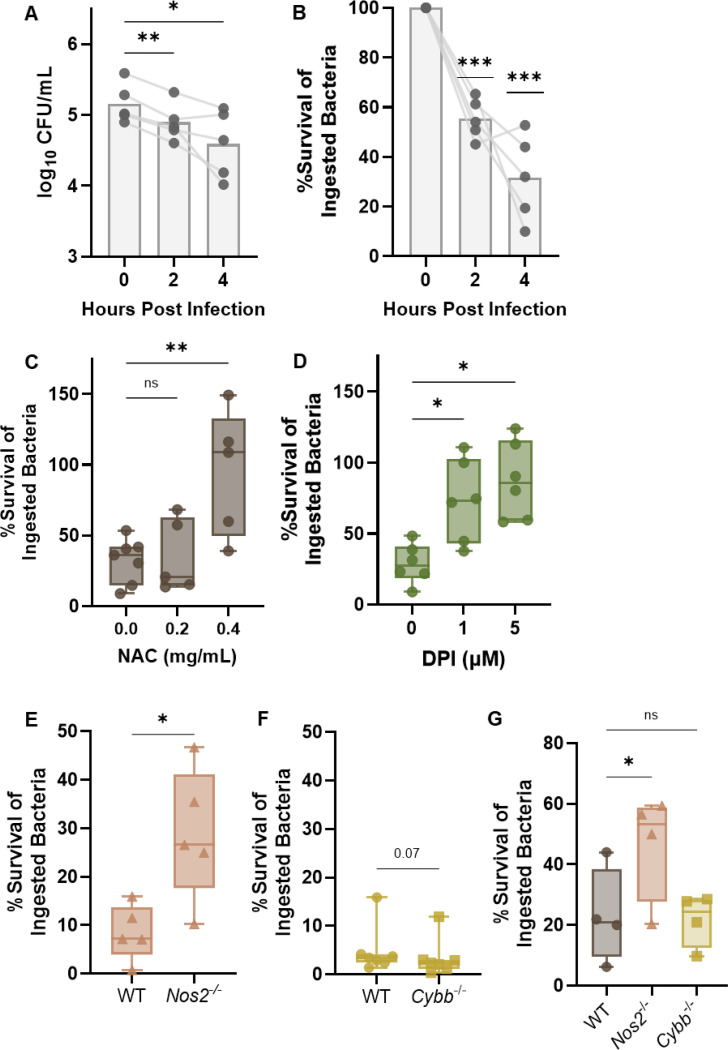
iNOS is required for effective macrophage clearance of *K. pneumoniae*. Macrophages were infected with KPPR1 for one hour prior to the removal of extracellular bacteria with gentamicin treatment. Abundance of intracellular bacteria was assessed immediately (T0), after two hours (T2), or after four hours (T4). In (A), the abundance of intracellular KPPR1 is displayed as log_10_ CFU/mL. *p<0.05, **p<0.01 by paired one-way ANOVA. In (B), the results from (A) are displayed as percent survival, calculated as: (T0 CFU/T2 or T4 CFU)*100. ***p<0.001 by one sample *t*-test with a hypothetical value of 100. (C) N-acetyl-cysteine (NAC) or (D) diphenyleneiodonium (DPI) were used to broadly inhibit reactive species or NOX2 and iNOS activity, respectively, at the indicated concentration prior to assessment of KPPR1 intracellular survival. For (C-D), *p<0.05, **p<0.01 by paired one-way ANOVA. Primary bone marrow-derived macrophages from WT, (E) *Nos2*^−/−^, or (F) *Cybb*^−/−^ mice were assessed for the ability to kill intracellular KPPR1. (G) The results for WT, *Nos2*^−/−^, and *Cybb*^−/−^ cells were validated using immortalized bone marrow-derived macrophages. For (E-F) *p<0.05 by paired *t*-test and *p* values <0.1 are displayed as text; for (G) *p<0.05 by paired one-way ANOVA comparing each mutant macrophage to WT. For all, n=4–7 trials with symbols representing independent experiments. For bar graphs in (A-B), the top of the bar presents the mean values. For box plots in (CG), box boundaries represent the 25^th^ and 75^th^ percentile ranges, the middle line represents the median value, and the whiskers represent the minimum and maximum values.

To characterize macrophage bactericidal mechanisms against *K. pneumoniae,* we first needed to determine the timeframe in which a significant amount of bacterial killing is present. Using gentamicin protection assays, immortalized bone marrow-derived macrophages (iBMDMs) were exposed to the hypervirulent strain KPPR1 for 1 hour (21). Extracellular bacteria were killed with gentamicin, an antibiotic that does not permeate eukaryotic membranes. Intracellular *K. pneumoniae* was either measured immediately (T0) or after two (T2) and four (T4) hours. Macrophages displayed significant killing of *K. pneumoniae* as early as two hours post infection, with further killing observed after four hours (Figure 1A–B).

Two prominent mechanisms used by macrophages to eliminate internalized bacteria are the production of ROS and RNS. While *K. pneumoniae* is susceptible to killing by both stressors (21), it is unknown whether macrophages specifically leverage ROS and RNS to kill intracellular *K. pneumoniae*. To determine the role of general reactive species in macrophage interactions with *K. pneumoniae*, we broadly neutralized these species by adding the antioxidant N-acetylcysteine (NAC) to the media of infected cells (22). At low NAC concentrations (0.2mg/mL), minimal alterations in macrophage killing were observed, but *K. pneumoniae* survival significantly increased at higher NAC concentrations (Figure 1C, Supplemental Figure 1A). NAC had minimal direct effects on *K. pneumoniae* viability as bacterial growth was observed when macrophages were absent (Supplemental Figure 1B). Next, we assessed whether inhibiting sources of ROS and RNS influenced macrophage killing of *K. pneumoniae.* Cells were treated with diphenyleneiodonium (DPI), a chemical inhibitor of flavoenzymes like the NOX2 complex and iNOS prior to infection (23, 24). *K. pneumoniae* survival was significantly increased after DPI treatment (Figure 1D, Supplemental Figure 1C). While DPI did not exhibit bactericidal properties over a four hour period, high concentrations dampened *K. pneumoniae* replication in media alone (Supplemental Figure 1D). Thus, macrophages leverage reactive species that likely originate from flavoenzymes to clear internalized *K. pneumoniae*.

To specifically test the involvement of macrophage NOX2 and iNOS in the clearance of *K. pneumoniae*, we generated primary BMDMs from WT mice and mice lacking the genes *Cybb* and *Nos2* (25). CYBB is a critical component of the NOX2 complex, and in its absence the phagocyte NADPH oxidase cannot assemble; *Nos2* encodes the iNOS enzyme. In the absence of *Nos2*, *K. pneumoniae* survival was significantly elevated compared to WT cells (Figure 1E, Supplemental Figure 2A). In contrast, no difference was observed in *K. pneumoniae* survival between WT cells and those lacking CYBB (NOX2), as both were highly effective at killing *K. pneumoniae* (Figure 1F, Supplemental Figure 2B). We validated these results using iBMDMs sourced from different mice and confirmed that *K. pneumoniae* intracellular survival is significantly increased in cells lacking iNOS (Figure 1G, Supplemental Figure 2C). Collectively, these data confirm that monocyte-derived macrophages leverage iNOS to kill intracellular *K. pneumoniae* and that the NADPH oxidase is dispensable for this process.

### iNOS2 is required for normal inflammatory signaling in the presence of *K. pneumoniae*.

**Figure 2.**
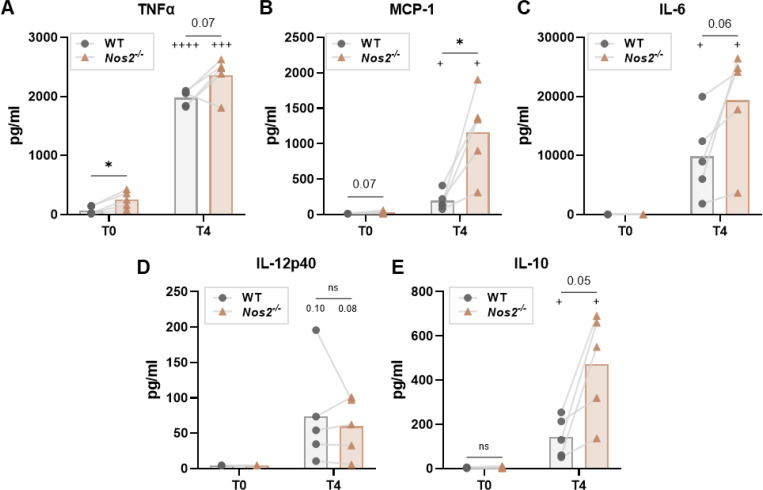
iNOS is required for normal cytokine production in the presence of *K. pneumoniae*. Wild-type and *Nos2*^−/−^ primary bone marrow-derived macrophages were infected with KPPR1 for one hour prior to the removal of extracellular bacteria with gentamicin. Supernatant was collected after gentamicin treatment (T0) or four hours post-infection (T4). Macrophage supernatants were evaluated for cytokine abundance using ELISAs for (A) TNFα, (B) MCP-1, (C) IL-6, (D) IL-12p40, and (E) IL-10. *p<0.05 by paired *t*-test; +*p*<0.05, +++*p*<0.001, ++++*p*<0.0001 by paired *t*-test comparing cytokine abundance at T4 to T0 for each cell type. For all, *p* values <0.1 are displayed as text; n=5 trials with symbols representing independent experiments. The top of each bar represents mean values.

To test whether normal *K. pneumoniae*-induced inflammation is dependent upon iNOS, we infected WT and *Nos2*^−/−^ primary BMDMs as in Figure 1E and collected supernatant at the beginning and end of the infection. Supernatants were subjected to ELISA to measure the abundance of cytokines produced in response to *K. pneumoniae.* Both WT and *Nos2*^−/−^ BMDMs had a significant increase in TNFα secretion during *K. pneumoniae* infection (Figure 2A). *Nos2*^−/−^ cells trended towards a higher TNFα response after four hours, but this may be due to elevated presence at baseline. *Nos2*^−/−^ cells displayed elevated release of the pro-inflammatory MCP-1 and IL-6 in response to *K. pneumoniae*, even though these cytokines were minimally detected at baseline (Figure 2B–C). However, we did not detect this across all pro-inflammatory cytokines. IL-12p40, the active subunit of IL-12 and IL-23, was modestly released in response to *K. pneumoniae* but there was no detectable difference between WT and *Nos2*^−/−^ cells (Figure 2D). IL-10 is considered an anti-inflammatory cytokine, but its expression is largely regulated by inflammatory responses (Figure 2E). Thus, we were not surprised that *Nos2*^−/−^ macrophages released more IL-10 during *K. pneumoniae* infection than WT cells. Neither WT or *Nos2*^−/−^ macrophages elicited IL-1β during these infections as all supernatants were below the limit of detection (data not shown). Collectively, *Nos2*^−/−^ macrophages could be less effective at killing *K. pneumoniae* due to a hyper-inflammatory response that obscures normal killing. The increased inflammation in *Nos2*^−/−^ cells may also be due to a higher abundance of internalized *K. pneumoniae* due to defects in intracellular killing in the absence of iNOS (Figure 1E, G). These data reveal that iNOS is responsible for normal cellular defense and cytokine release by macrophages in response to *K. pneumoniae*. Thus, iNOS may contribute to *K. pneumoniae* clearance through mechanisms linked to both RNS production and normal immune orchestration.

### Macrophage ROS bursts are not detectable in response to intracellular *K. pneumoniae*.

**Figure 3.**
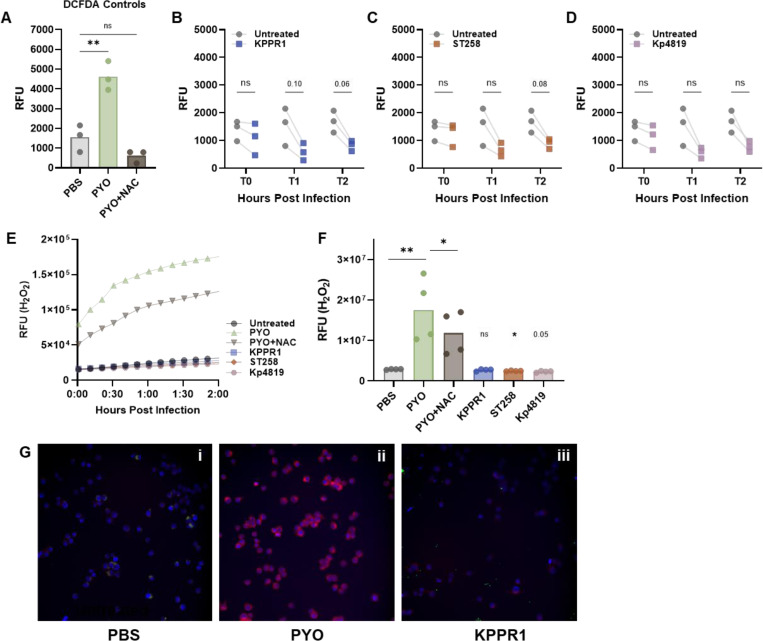
Monocyte-derived macrophages elicit no detectable ROS burst in response to *K. pneumoniae*. (A) Immortalized bone marrow derived macrophages were untreated (PBS), exposed to pyocyanin, or treated with pyocyanin+N-Acetyl L-Cysteine (NAC) prior to DCFDA staining. Relative fluorescence (RFU) of controls was measured 1 hour post infection. Macrophages were infected with (B) KPPR1, (C) ST258, or (D) 4819 and stained with DCFDA. (E) Macrophages were treated as above prior to AmplexRed staining. RFU was measured every 10 minutes for two hours, and the average RFU for four trials is displayed. (F) Area under the curve (AUC) was measured for the data in (E). (G) Macrophages were untreated (i), treated with pyocyanin (ii), or infected with KPPR1-chromoGFP (iii) then stained with CellROX (red) and NucBlue (blue). Representative images are displayed for one of three trials. For (A) **p<0.01 by one-way ANOVA; for (B-D), no comparisons were statistically significant by paired *t*-test and lines connect data from the same trial. For (F), comparisons with lines are paired *t*-tests comparing the experimental control groups and *p<0.05, **p<0.01; the AUC for cells infected with KPPR1, ST258, and Kp4819 were compared to untreated cells using a paired one-way ANOVA and *p<0.05. For all panels, comparisons with *p*<0.1 are indicated in text and n=3–4 independent trials. In (A) and (F), the tops of bars represent mean values; in all graphs, symbols represent independent trials.

We were surprised to observe that NOX2 was dispensable for the intracellular killing of *K. pneumoniae* (Figure 1F–G) given that this is a potent anti-bacterial effector used for many different pathogens (20, 26). Therefore, we measured the extent to which *K. pneumoniae*-infected macrophages generate a ROS burst. *K. pneumoniae* is a remarkably diverse species consisting of two major pathotypes, hypervirulent and classical, which can vary in their interactions with the host (13, 27). We assessed the hypervirulent strain KPPR1 and two classical strains: NJST258_2 (ST258) which contains a carbapenem resistance plasmid (28), and Kp4819 which was isolated from the gut of a hospitalized patient (29). First, broad reactive oxygen radicals were measured with the probe 2',7'-dichlorofluorescin diacetate (DCFDA), a fluorescent dye that tags intracellular redox species. Untreated iBMDMs had minimal ROS at baseline, and ROS bursts were detectable when cells were treated with pyocyanin, a phenazine which stimulates ROS (30) (Figure 3A). As a separate control, the signal resulting from pyocyanin treatment was confirmed to result from reactive species as it could be quenched with the anti-oxidant NAC. These controls indicate that cells are behaving as expected and were run with every ROS detection experiment.

Macrophage ROS was measured via DCFDA after cells were infected with KPPR1 (Figure 3B), ST258 (Figure 3C), and Kp4819 (Figure 3D). ROS signal was measured shortly after infection (T0), one (T1), or two hours post-infection (T2) since macrophage anti-bacterial effectors are significantly present at this time (Figure 1A–B). Macrophage ROS was undetectable in response to each *K. pneumoniae* strain. Instead, *K. pneumoniae* infected macrophages seemed to exhibit trends towards lower ROS than untreated cells, although this did not reach statistical significance. Thus, macrophages do not elicit an abundant ROS burst in response to *K. pneumoniae*. These results are supported by the finding that NOX2 activity is dispensable for the eradication of intracellular *K. pneumoniae* (Figure 1F–G). We then assessed whether this phenotype was specific to *K. pneumoniae* and measured ROS in macrophages infected with two other Gram-negative species, *Escherichia coli* or *Pseudomonas aeruginosa*. Macrophages generated detectable ROS bursts in response to infections with these species (Supplemental Figure 3A-B).

Next, we wanted to confirm the DCFDA results using a secondary probe, AmplexRed, which measures the accumulation of extracellular hydrogen peroxide (31). ROS detection via AmplexRed was feasible as macrophages stimulated with pyocyanin elicited a significantly higher signal than untreated cells, and the signal was quenched with NAC (Figure 3E–F). As with DCFDA, macrophages did not elicit detectable hydrogen peroxide release over a two hour period in response to any *K. pneumoniae* strain. Again, *K. pneumoniae* strains had seemingly lower ROS signal than untreated cells, and this reached statistical significance for the strain ST258 when measured with AmplexRed (Figure 3F).

We next visualized whether individual macrophages elicit ROS that would be undetectable within a population-based, plate reader assay like DCFDA or AmplexRed. Using the probe CellROX, which generates a fluorescent signal when in contact with hydroxyl species, we visualized ROS within KPPR1-infected cells. Untreated macrophages elicited no detectable ROS signal, but ROS could be visualized after pyocyanin treatment (Figure 3Gi–ii). Macrophages infected with KPPR1 demonstrated no detectable ROS signal on an individual cellular level (Figure 3Giii). These collective data using three independent techniques reveal that macrophages infected with *K. pneumoniae* do not elicit a detectable oxidative burst.

### *K. pneumoniae* does not inactivate NOX2 signaling.

**Figure 4.**
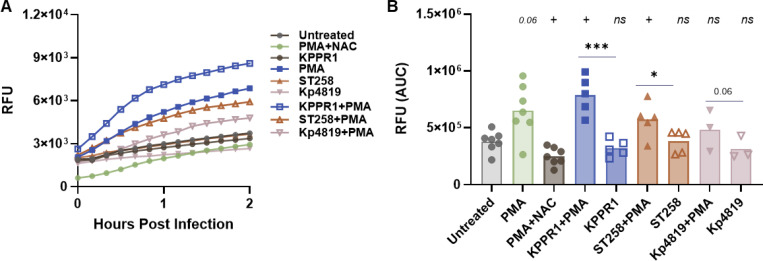
*K. pneumoniae* does not block induced NADPH signaling. Primary bone marrow derived macrophages were infected with KPPR1, ST258, or Kp4819 and either left alone or then stimulated with PMA. (A) The DCFDA relative fluorescence (RFU) of each well, including controls (untreated, PMA, and PMA+NAC), was measured every ten minutes for two hours. The mean value is displayed for each group. (B) Area under the curve (AUC) analysis was measured for the curves displayed in (A). For (B), +*p*<0.05 by one-way ANOVA comparing each group to the untreated control (UN); *p<0.05 and ***p<0.001 by paired *t*-test comparing the signal from macrophages infected with each *Kp* strain with and without a PMA stimulus. The top of each bar represents the mean RFU group value, n=4–7 and symbols represent independent trials.

Since infected macrophages did not elicit a detectable ROS burst, we tested whether this was due to *K. pneumoniae* inhibition of the active, NOX2 signaling cascade. First, macrophages were infected with *K. pneumoniae* for one hour to allow time for host-pathogen interactions to initiate. Then, NOX2 activity was induced using the protein-kinase-C (PKC) isotype activator phorbol 12-myristate 13-acetate (PMA). PKC activates *p*47^*phox*^, causing rapid assembly and induction of the NADPH oxidase upon stimulation (32, 33). We reasoned that if *K. pneumoniae* inhibited NOX2 activity, then infected macrophages would have minimal ROS signal after PMA treatment. Uninfected macrophages displayed elevated ROS signal after PMA treatment and NAC quenched the signal (Figure 4A–B). As before, no ROS signal was detected in cells treated with *K. pneumoniae* alone (for KPPR1, ST258, or KP4819). However, infected macrophages which were treated with PMA did indeed produce a substantial ROS burst. While cells infected with Kp4819 demonstrated no significant difference in ROS signal after PMA treatment, there was a strong trend towards higher signal compared to Kp4819-infected that did not receive PMA. Collectively, these data indicate that NOX2 assembly and activity can still occur in the presence of *K. pneumoniae*. Instead, the lack of macrophage ROS in response to *K. pneumoniae* is likely due to prevention of NADPH assembly at earlier stages of infection.

### *K. pneumoniae* evades ROS generation in alveolar-like macrophages.

**Figure 5.**
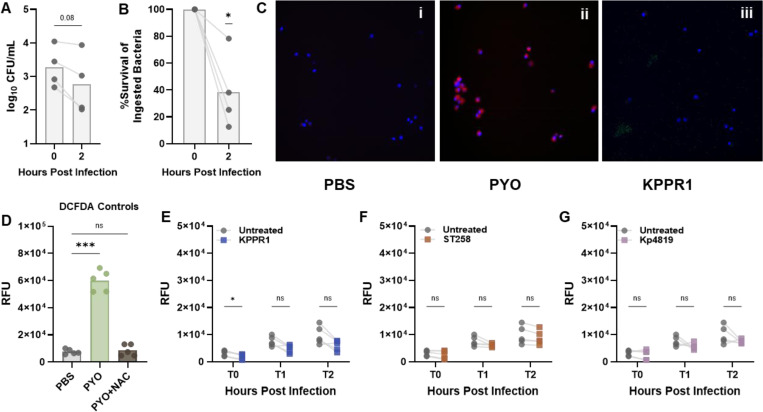
Alveolar-like macrophages elicit no detectable ROS burst in response to *K. pneumoniae*. Fetal liver-derived alveolar-like macrophages (FLAMs) were infected with KPPR1 for one hour prior to the removal of extracellular bacteria with gentamicin treatment. The abundance of intracellular bacteria was assessed immediately (T0) or after two hours (T2). In (A), the abundance of intracellular KPPR1 is displayed as log_10_ CFU/mL with significance assessed by paired *t*-test and the *p*-value indicated by text. In (B), the results from (A) are displayed as percent survival, calculated as: (T0 CFU/T2 CFU)*100. *p<0.05 by one sample *t*-test with a hypothetical value of 100. (C) FLAMs were untreated (i), treated with pyocyanin (ii), or infected with KPPR1-chromoGFP (green, iii) then stained with CellROX (red) and NucBlue (blue). Representative images are displayed for one of three independent trials. (D) FLAMs were left untreated, exposed to pyocyanin, or treated with pyocyanin+N-Acetyl L-Cysteine prior to DCFDA staining. Relative fluorescence (RFU) of controls was measured 1 hour post infection. ***p<0.001 by one way ANOVA. FLAMs were infected with (E) KPPR1, (F) ST258, or (G) Kp4819 and stained with DCFDA. *p<0.05 by paired *t*-test and lines connect values from independent trials. For all, symbols represent independent trials; in (A), (B), and (D), the tops of bars indicate mean values.

Since ROS generation was undetectable in *K. pneumoniae*-infected monocyte-derived cells, we tested whether this was consistent across other macrophage lineages. Both monocyte-derived and alveolar macrophage lineages are important for *K. pneumoniae* infection control, yet have fundamentally different host-pathogen interactions as they arise from different progenitors and have distinct baseline functions (14–16, 34, 35). To investigate whether *K. pneumoniae*-infected alveolar-like macrophages lack an ROS burst, we leveraged an *ex vivo* model of fetal liver-derived alveolar-like macrophages (FLAMs), (35). FLAMs were less phagocytic against KPPR1 than BMDMs, but demonstrated significant bactericidal activity after two hours (Figure 5A–B). FLAMs were infected with KPPR1 and ROS was visualized with CellROX as in Figure 3. Untreated FLAMs displayed minimal ROS in comparison to the abundant ROS signal in pyocyanin-treated FLAMs (Figure 5Ci–ii). As with BMDMS, KPPR1-infected FLAMs had no detectable ROS burst via microscopy (Figure 5Ciii). These data were confirmed with DCFDA. FLAMs were capable of generating a robust ROS burst as treatment with pyocyanin resulted in a significantly higher DCFDA signal than untreated cells or cells where the pyocyanin signal was quenched with NAC (Figure 5D). Again, no ROS signal was detected across KPPR1 (Figure 5E), ST258 (Figure 5F), or Kp4819 (Figure 5G). These data confirm two distinct macrophage lineages do not elicit detectable ROS upon infection with diverse strains of *K. pneumoniae.*

## Discussion

*K. pneumoniae* is a clinically relevant pathogen of urgent concern, but surprisingly little is known about its host-pathogen interactions. While macrophages significantly enhance *K. pneumoniae* clearance during infection, the cellular mechanisms used to eliminate this pathogen remain minimally described. Here, we tested the contributions of two macrophage anti-bacterial effector mechanisms, oxidative and nitrosative stress, to the killing of *K. pneumoniae*. Our data reveal that macrophages utilize iNOS, rather than the phagocyte NADPH oxidase NOX2, as a *K. pneumoniae* clearance mechanism (Figure 1). iNOS likely supports *K. pneumoniae* elimination both through RNS production and orchestrating a normal immune response (Figure 2). Macrophages infected with *K. pneumoniae* demonstrated no detectable ROS burst, further supporting the dispensability of NOX2 in these interactions (Figure 3). *K. pneumoniae* likely blocks NOX2 initiation, rather than interfering with signaling once it occurs (Figure 4). Importantly, these findings are applicable across macrophage lineages and *K. pneumoniae* strains (Figure 5), highlighting novel host-pathogen interactions that are applicable in multiple infection types.

It is well-established that NOX2 is critical for host defense against *K. pneumoniae*. Our research group and others have reported that mice lacking functional NOX2 have higher bacterial burdens in the lung and lower survival following *K. pneumoniae* infection (36–38). Mice lacking NOX2 also have altered *K. pneumoniae* dissemination patterns from the lung to the blood, seemingly due to ROS-dependent control of the initial KPPR1 founding population (36). However, ROS is not equally effective in anti-*K. pneumoniae* defense across immune subsets. A previous study found that the NOX2 inducing protein SKAP2 is required in neutrophils, not macrophages, to support KPPR1 clearance *in vivo* (38). In accordance with this study, our data show that macrophages do not require ROS to kill intracellular *K. pneumoniae.* Thus, neutrophils are likely the most prominent source of oxidative stress during *K. pneumoniae* infections.

Instead, our data reveal that iNOS is required for effective macrophage control of *K. pneumoniae* (Figure 1E, G). On average, BMDMs derived from *Nos2*^−/−^ mice had ~5 fold lower KPPR1 killing than WT cells. iNOS is also required for *in vivo* defense against KPPR1, as mice treated with the iNOS inhibitor L-NAME had significantly lower survival and elevated bacterial burden in the lung and blood (39). Alveolar macrophages treated with L-NAME demonstrate reduced *K. pneumoniae* killing, demonstrating that this mechanism is used across multiple macrophage lineages (39). We also identified roles for iNOS in defense against *K. pneumoniae* beyond direct bactericidal activity as cytokine production was altered in the absence of iNOS. Our data aligns with previous reports that iNOS partially dampens M1 polarization, such that cells lacking iNOS may be more likely to transition to an inflammatory state (40).

*K. pneumoniae* bacteremia fitness factors have multiple overlapping roles with the ability to resist oxidative, nitrosative, and macrophage-mediated stress (21). The extent to which bacteremia fitness mutants resist RNS is significantly correlated with their ability to resist macrophage-mediated stress, yet there is no substantial link between resistance against oxidative stress and resistance to the intracellular environment. Our results in the present study support these findings. The contributions of iNOS, and the dispensability of NOX2, further define that macrophages leverage RNS to elicit stress on intracellular *K. pneumoniae*. Nitric oxide can have significant impacts on *K. pneumoniae,* including the alteration of capsule and biofilm formation (41). *K. pneumoniae* also generates inherent RNS in response to external oxidative and nitrosative stress (21), meaning that these stressors could potentiate bacterial death by eliciting self-production of this molecule. Accordingly, *K. pneumoniae* employs multiple mechanisms to resist nitrosative stress. Various *K. pneumoniae* regulators and metabolic genes enhance resistance against RNS, although studies identifying these RNS resistance factors have tested a limited number of factors (21, 42). Further work should identify the broader scope of *K. pneumoniae* genes regulating resistance against nitrosative stress as targeting these pathways could support more effective bacterial clearance by macrophages.

In support of the finding that NOX2 is dispensable for macrophage clearance of *K. pneumoniae*, we identified no detectable ROS burst within infected cells. Our monocyte-derived and alveolar-like macrophages displayed healthy morphology and bactericidal capabilities (Figure 3, Figure 5). We were also able to detect ROS within both cell types through positive controls, confirming that the cells were capable of producing ROS bursts. However, infected cells from neither lineage elicited a detectable ROS signature. Since *K. pneumoniae* did not dampen ROS when initiated via PMA treatment, we believe that initial NOX2 assembly within macrophages is prevented rather than *K. pneumoniae* playing an active role in inhibiting ROS signaling.

The absence of ROS within macrophages infected with diverse isolates indicates that *K. pneumoniae* has conserved mechanisms for thwarting this host defense (Figures 3–5). As a species, *K. pneumoniae* is composed of two major pathotypes containing hypervirulent and classical strains. Hypervirulent strains are characterized by enhanced capsule production, siderophores, and invasive disease in healthy patients (43). Classical strains are associated with multidrug resistance and outbreaks in healthcare settings. These differences can alter interactions with the host including bacterial recognition, phagocytosis, and induced inflammation (13, 44). To account for these differences, our study incorporated three diverse isolates, one hypervirulent (KPPR1) and two classical (ST258, Kp4819) strains. As no strain of *K. pneumoniae* in this study elicited macrophage ROS across two distinct lineages, our results suggest a widely applicable phenotype of ROS blocking by this pathogen.

We recognize that our study has limitations including the use of *in vitro* assays, the use of one macrophage state, and the investigation of one immune subset. Within infected tissue, *K. pneumoniae* is exposed to a cytokine-rich environment and other cell-cell interactions that are not accounted for with our *in vitro* approach (45). However, we believe the current approach is sufficient to detect distinct contributions from NOX2 and iNOS in this process. While our work focused on murine macrophages, it is possible that iNOS has differential contributions within human cells. Although M1 or M2 polarization may influence *K. pneumoniae* clearance, we observed substantial markers of activation via cytokine production by infecting unpolarized cells (21). While our study was focused on the role of NOX2 and iNOS in macrophage killing of *K. pneumoniae*, it did not extend these characterizations to other innate immune subsets such as neutrophils. NOX2 is an established source of anti-*K. pneumoniae* defense by neutrophils, but the extent to which neutrophil iNOS supports *K. pneumoniae* killing will be the focus of future work.

Collectively, our study provides new insight into the mechanisms used by macrophages to eliminate intracellular *K. pneumoniae*. We discovered surprising, divergent roles for NOX2 and iNOS in macrophage defense against *K. pneumoniae* with iNOS being required for effective killing of intracellular *K. pneumoniae*. Thus, pathways upstream of iNOS that elicit this response may be highly relevant for enhancing host defense against this dangerous pathogen.

## Materials and Methods

### Bacterial Strains and Materials.

All materials and reagents were sourced from ThermoFisher (Fisher Scientific, Waltham, MA) unless stated otherwise. The *K. pneumoniae* strains used in this study are: ATCC 43816 (KPPR1), 30684/NJST258_2 (ST258), and Kp4819 (28, 46, 47). The fluorescent strain KPPR1-chromoGFP expresses constitutive GFP expression from the chromosome and was constructed previously (21). *K. pneumoniae* strains were stored at −80°C and maintained on Lysogeny broth (LB, BP1425500) plates for up to 3 weeks at 4°C. Prior to experiments, bacteria were cultured by inoculating 2 mL of LB broth (BP1426500) with a single colony and grown overnight, shaking at 37°C. *K. pneumoniae* overnight cultures were washed with phosphate-buffered saline (PBS, MT21040CV) and adjusted to the appropriate experimental concentration based on OD_600_ density. Quantitative culture was used to verify the bacterial input for all experiments.

### Cell Culture and Primary Cell Isolation

Immortalized bone marrow derived macrophages (iBMDMs) were initially derived from C57BL/6 mice (48, 49). iBMDMs were cultured using DMEM+10% heat-inactivated fetal bovine serum (FBS; MT35011CV)+1% penicillin-streptomycin (MT30009CI) on 100mm tissue culture treated dishes (FB012924) until 60–90% confluency. For passaging, cells were rinsed with PBS and lifted from the dish using with 2mM ethylenediaminetetraacetic acid (EDTA; BP2482100). Cells were washed, resuspended in DMEM (MT10017CV)+10% FBS, and quantified using a hemacytometer with viability assessed by trypan blue (15-250-061) exclusion. All cells used in this study demonstrated >90% viability. Once quantified, cells were either seeded for experimentation or passaged for continued proliferation. iBMDMs used for experiments were between 5–15 passages.

Primary BMDMs (pBMDMs) were isolated from the femurs of mice 6–18 weeks. Bone marrow was flushed with DMEM+10% heat inactivated FBS+1% penicillin-streptomycin and passed through a 40 micron cell strainer (08-771-1). Cells were washed and resuspended in DMEM+10% heat inactivated FBS+1% penicillin-streptomycin+15% L-cell supernatant. Nucleated cells were counted on a hemacytometer and seeded into non-tissue culture treated 100 mm dishes at a density of 5 million cells (FB0875712). 3–4 days after initial seeding the media was exchanged. One week after seeding and subsequent proliferation, pBMDMs were harvested by lifting with 2mM EDTA. The cell suspensions were pelleted and resuspended in fresh media prior to quantification. Frozen pBMDM stocks were prepared by aliquoting cells into cryo-grade vials with 10% dimethyl sulfoxide (DMSO; 25-950-CQC). Stocks were placed into foam containers to allow for gradual cooling and moved 24 hours later to liquid nitrogen for long term storage. Once thawed from liquid nitrogen, pBMDMs were seeded for experimentation.

Fetal liver-derived cells were isolated as previously described by plating cells retrieved from the livers from mice at roughly E18 (50). Alveolar-like macrophages (FLAMs) were then generated as previously detailed (35). FLAMs were cultured in 100mm tissue culture dishes in RPMI (MT10040CV)+10% heat inactivated FBS+1% penicillin streptomycin+30ng/ml recombinant GM-CSF+15ng/ml recombinant TGF*β* (PeproTech, Cranbury, NJ: 3150320UG, 100-21-10UG). FLAMs were seeded at densities no less than 750,000 and no more than 2 million cells per dish and passaged at 60–75% confluency. Cells were lifted from the dishes using 10mM EDTA, washed, and enumerated on a hemacytometer. FLAMs used for experiments were between 5–12 passages.

### Gentamicin Protection Assays.

To assess *K. pneumoniae* uptake and intracellular survival within macrophages, gentamicin protection assays were performed as previously described (21). Briefly, iBMDMs were seeded and infected with *K. pneumoniae* at a target ratio of 10 bacteria per cell (MOI 10) in 24 well tissue culture treated dishes (09-761-146). The infection was synchronized by centrifugation at 500xg for five minutes at 4°C. The plate was then incubated at 37°C with 5% CO_2_ for one hour to allow for bacterial uptake. Extracellular bacteria was killed with gentamicin (15750060) at a concentration of 100ug/mL in the appropriate cell culture media for 30 minutes. Afterwards, a subset of wells were washed with 1mL PBS and lysed with 500uL of filter-sterilized 1x saponin (AAJ63209AK), and bacterial uptake assessed by quantitative culture (T0). A separate set of wells were incubated for an additional 2 or 4 hours at 37°C, 5% CO_2_ prior to washing, lysis, and enumeration (T2, T4). Intracellular survival was assessed as the abundance of *K. pneumoniae* surviving at each timepoint: percent survival of ingested bacteria = (CFU T2 or T4/CFU T0)*100. Experiments using chemical inhibitors had the following modifications. Nacetylcysteine (NAC, 20261; Cayman Chemical Company, Ann Arbor, MI) was diluted into cell culture media and added to macrophages at the time of infection at 0.2 or 0.4mg/mL. For diphenyleneiodonium chloride (DPI, 50-176-2440; Sigma-Aldrich, St. Louis, MO), 1 or 5μM/mL was added to macrophages at the time of initial cell seeding, roughly 16 hours prior to the start of the experiment. For all experiments using chemical inhibitors, the ability of *K. pneumoniae* to replicate in the presence of the inhibitor was assessed. *K. pneumoniae* (1×10^7^ CFU) was added to media containing individual inhibitors at the concentrations listed above. Bacterial survival was quantified by sampling wells for enumeration at T0 and T4 (Supplemental Figure 1). For gentamicin protection assays using FLAMs, macrophages were seeded in tissue culture treated 12 well dishes using RPMI+10%FBS. For experiments using primary BMDMs, cells were seeded in DMEM+10% FBS in non-tissue culture treated 6 well dishes.

### ELISA.

The supernatants of infected pBMDMs were analyzed for cytokine abundance using ELISA. Macrophages were infected as detailed for gentamicin protection assays. At the T0 and T4 timepoint, 1mL of media was removed and immediately banked at −80°C. Supernatants remained frozen until analysis by the University of Minnesota Cytokine Reference Laboratory. All ELISAs were performed with standard curves and positive and negative controls.

### Measurement of ROS.

All ROS detection assays included controls to assess baseline ROS fluorescence (PBS, untreated cells) and baseline *K. pneumoniae* fluorescence (bacteria only). Assays also included positive controls to ensure ROS signal was detectable, either 1mM pyocyanin (601522) or 1.6mM phorbol 12-myristate 13-acetate (PMA, 50-288-7125; Cayman Chemical Company, Ann Arbor, MI) depending on the assay.

To measure intracellular radicals, 2',7'-dichlorofluorescin diacetate (DCFDA) was used (Cayman Chemical; 601520). Macrophages were seeded in a black-walled tissue-culture treated 96-well dish (353219) at 5×10^4^ cells/well in the appropriate cell culture media and infected with *K. pneumoniae* to achieve MOI 50. After infection, cells were centrifuged at 500xg for 5 minutes to synchronize infection and incubated at 37°C, 5% CO_2_ for 1 hour. DCFDA staining occurred following the manufacturer's protocol with the exception that 100ug/mL of gentamicin was added to ROS Staining Buffer to kill extracellular bacteria. After the 30-minute staining incubation, fluorescence was measured using the Synergy H1 Hybrid plate reader with an excitation wavelength of 490nm and an emission wavelength of 530nm. Relative fluorescence units (RFU) was calculated by averaging two technical replicates of infected cells then subtracting background signal generated by bacteria using fluorescence from wells containing bacteria only (no cells). For all *K. pneumoniae* strains, this signal was minimal to undetectable.

For AmplexRed assays, BMDMs were seeded in black-walled tissue-culture treated 96-well dishes at 5×10^4^ cells per well. Macrophages were infected with *K. pneumoniae* with a target MOI of 50. After infection, cells were centrifuged at 500xg for 5 minutes to synchronize infection and incubated at 37°C, 5% CO_2_ for 1 hour. Cells were washed and Amplex Red stock solution was applied (10mM Amplex Red Reagent, A22188, 10U/mL HRP stock solution, 1X Reaction Buffer) to each well. Technical replicates were run in duplicate and values were averaged. Fluorescence was measured every 10 minutes for 2 hours, with emission detection at 590nm and an absorbance of 560nm using a Synergy H1 Hybrid plate reader.

For CellROX imaging, macrophages were seeded in black-walled tissue-culture treated 96-well dishes at 1×10^4^ cells per well in the appropriate cell culture media. Cells were either uninfected, treated with 1mM pyocyanin or infected at an MOI 50 with KPPR1-chromoGFP. Plates were centrifuged at 500xg for 5 minutes to facilitate infection and incubated at 37°C, 5% CO_2._ After incubation, media was removed, and cells were treated with 100ug/mL gentamicin for 30 minutes. Cells were then washed, stained with CellROX Red (C10422) and NucBlue (PI62249) for 30 minutes, and fixed with 4% paraformaldehyde (15714). Cells were imaged within an hour of staining utilizing 20x magnification on a Keyence BZ-X810 fluorescence microscope. Technical replicates were run in duplicate to ensure similar patterns in stained could be observed across wells.

### Statistical Analysis.

Statistical significance was calculated using GraphPad Prism software. Ordinary one-way ANOVA with Dunnett’s multiple comparisons was used to define differences in means among multiple groups in reference to a single mean, one-sample *t*-tests calculated the difference in a group in reference to a theoretical mean, paired or unpaired *t*-tests compared differences among two groups. A *p*-value <0.05 was determined to be statistically significant. All assays were run in at least three independent trials.

## Supplementary Material

Supplement 1

## Data Availability

Data underlying all figures are included as individual data points within main and supplemental figures.

## References

[1] Singer M, Deutschman CS, Seymour CW, Shankar-Hari M, Annane D, Bauer M, The Third International Consensus Definitions for Sepsis and Septic Shock (Sepsis-3). JAMA. 2016;315(8):801–10. 26903338 10.1001/jama.2016.0287 PMC4968574

[2] Hajj J, Blaine N, Salavaci J, Jacoby D. The “Centrality of Sepsis”: A Review on Incidence, Mortality, and Cost of Care. Healthcare (Basel). 2018;6(3):90. 30061497 10.3390/healthcare6030090 PMC6164723

[3] Novosad SA, Sapiano MR, Grigg C, Lake J, Robyn M, Dumyati G, Vital Signs: Epidemiology of Sepsis: Prevalence of Health Care Factors and Opportunities for Prevention. MMWR Morb Mortal Wkly Rep. 2016;65(33):864–9. 27559759 10.15585/mmwr.mm6533e1

[4] Diekema DJ, Hsueh PR, Mendes RE, Pfaller MA, Rolston KV, Sader HS, The Microbiology of Bloodstream Infection: 20-Year Trends from the SENTRY Antimicrobial Surveillance Program. Antimicrob Agents Chemother. 2019;63(7):e00355–19. 10.1128/AAC.00355-19 PMC6591610 31010862

[5] Wisplinghoff H, Bischoff T, Tallent SM, Seifert H, Wenzel RP, Edmond MB. Nosocomial bloodstream infections in US hospitals: analysis of 24,179 cases from a prospective nationwide surveillance study. Clin Infect Dis. 2004;39(3):309–17. 15306996 10.1086/421946

[6] Magill SS, Edwards JR, Bamberg W, Beldavs ZG, Dumyati G, Kainer MA, Multistate point-prevalence survey of health care-associated infections. N Engl J Med. 2014;370(13):1198–208. 24670166 10.1056/NEJMoa1306801 PMC4648343

[7] Holmes CL, Anderson MT, Mobley HLT, Bachman MA. Pathogenesis of Gram-Negative Bacteremia. Clin Microbiol Rev. 2021;34(2). 10.1128/CMR.00234-20 PMC8549824 33692149

[8] Vincent JL, Rello J, Marshall J, Silva E, Anzueto A, Martin CD, International study of the prevalence and outcomes of infection in intensive care units. JAMA. 2009;302(21):2323–9. 19952319 10.1001/jama.2009.1754

[9] Holmes CL, Albin OR, Mobley HLT, Bachman MA. Bloodstream infections: mechanisms of pathogenesis and opportunities for intervention. Nat Rev Microbiol. 2024. 10.1038/s41579-024-01105-2 PMC12519459 39420097

[10] Collaborators AR. Global burden of bacterial antimicrobial resistance in 2019: a systematic analysis. Lancet. 2022;399(10325):629–55. 35065702 10.1016/S0140-6736(21)02724-0 PMC8841637

[11] Sati H, Carrara E, Savoldi A, Hansen P, Garlasco J, Campagnaro E, The WHO Bacterial Priority Pathogens List 2024: a prioritisation study to guide research, development, and public health strategies against antimicrobial resistance. Lancet Infect Dis. 2025. 10.1016/S1473-3099(25)00118-5 PMC12367593 40245910

[12] Holmes CL, Smith SN, Gurczynski SJ, Severin GB, Unverdorben LV, Vornhagen J, The ADP-Heptose Biosynthesis Enzyme GmhB is a Conserved Gram-Negative Bacteremia Fitness Factor. Infect Immun. 2022;90(7):e0022422. 10.1128/iai.00224-22 PMC9302095 35762751

[13] Wong Fok Lung T, Charytonowicz D, Beaumont KG, Shah SS, Sridhar SH, Gorrie CL, *Klebsiella pneumoniae* induces host metabolic stress that promotes tolerance to pulmonary infection. Cell Metab. 2022;34(5):761–74.e9. 35413274 10.1016/j.cmet.2022.03.009 PMC9081115

[14] Xiong H, Carter RA, Leiner IM, Tang YW, Chen L, Kreiswirth BN, Distinct Contributions of Neutrophils and CCR2+ Monocytes to Pulmonary Clearance of Different *Klebsiella pneumoniae* Strains. Infect Immun. 2015;83(9):3418–27. 26056382 10.1128/IAI.00678-15 PMC4534658

[15] Chen L, Zhang Z, Barletta KE, Burdick MD, Mehrad B. Heterogeneity of lung mononuclear phagocytes during pneumonia: contribution of chemokine receptors. Am J Physiol Lung Cell Mol Physiol. 2013;305(10):L702–11. 24056971 10.1152/ajplung.00194.2013 PMC3840272

[16] Broug-Holub E, Toews GB, van Iwaarden JF, Strieter RM, Kunkel SL, Paine R, Alveolar macrophages are required for protective pulmonary defenses in murine *Klebsiella pneumonia*: elimination of alveolar macrophages increases neutrophil recruitment but decreases bacterial clearance and survival. Infect Immun. 1997;65(4):1139–46. 9119443 10.1128/iai.65.4.1139-1146.1997 PMC175109

[17] Janeway C, Travers P, Walport M, Shlomchik M. The Induced Responses of Innate Immunity. Immunobiology: The Immune System in Health and Disease. 9th ed. New York: Garland Science; 2017.

[18] Nguyen JA, Yates RM. Better Together: Current Insights Into Phagosome-Lysosome Fusion. Front Immunol. 2021;12:636078. 10.3389/fimmu.2021.636078 PMC7946854 33717183

[19] Vazquez-Torres A, Jones-Carson J, Mastroeni P, Ischiropoulos H, Fang FC. Antimicrobial actions of the NADPH phagocyte oxidase and inducible nitric oxide synthase in experimental salmonellosis. I. Effects on microbial killing by activated peritoneal macrophages in vitro. J Exp Med. 2000;192(2):227–36. 10899909 10.1084/jem.192.2.227 PMC2193262

[20] Fang FC. Antimicrobial reactive oxygen and nitrogen species: concepts and controversies. Nat Rev Microbiol. 2004;2(10):820–32. 15378046 10.1038/nrmicro1004

[21] Wilcox AE, Andres CJ, Bachman MA, Holmes CL. *Klebsiella pneumoniae* factors enhancing bacteremia have distinct contributions to macrophage-mediated, oxidative, and nitrosative stress resistance. Infect Immun. 2026:e0073925. 10.1128/iai.00739-25 PMC13081721 41728977

[22] Gillissen A, Nowak D. Characterization of N-acetylcysteine and ambroxol in anti-oxidant therapy. Respir Med. 1998;92(4):609–23. 9659525 10.1016/s0954-6111(98)90506-6

[23] Hampton MB, Winterbourn CC. Modification of neutrophil oxidant production with diphenyleneiodonium and its effect on bacterial killing. Free Radic Biol Med. 1995;18(4):633–9. 7750787 10.1016/0891-5849(94)00181-i

[24] Stuehr DJ, Fasehun OA, Kwon NS, Gross SS, Gonzalez JA, Levi R, Inhibition of macrophage and endothelial cell nitric oxide synthase by diphenyleneiodonium and its analogs. FASEB J. 1991;5(1):98–103. 1703974 10.1096/fasebj.5.1.1703974

[25] Werner JL, Escolero SG, Hewlett JT, Mak TN, Williams BP, Eishi Y, Induction of Pulmonary Granuloma Formation by Propionibacterium acnes Is Regulated by MyD88 and Nox2. Am J Respir Cell Mol Biol. 2017;56(1):121–30. 27607191 10.1165/rcmb.2016-0035OC PMC5248957

[26] Vatansever F, de Melo WC, Avci P, Vecchio D, Sadasivam M, Gupta A, Antimicrobial strategies centered around reactive oxygen species--bactericidal antibiotics, photodynamic therapy, and beyond. FEMS Microbiol Rev. 2013;37(6):955–89. 23802986 10.1111/1574-6976.12026 PMC3791156

[27] Russo TA, Alvarado CL, Davies CJ, Drayer ZJ, Carlino-MacDonald U, Hutson A, Differentiation of hypervirulent and classical *Klebsiella pneumoniae* with acquired drug resistance. mBio. 2024;15(2):e0286723. 10.1128/mbio.02867-23 PMC10865842 38231533

[28] Snitkin ES, Zelazny AM, Thomas PJ, Stock F, Henderson DK, Palmore TN, Tracking a hospital outbreak of carbapenem-resistant *Klebsiella pneumoniae* with whole-genome sequencing. Sci Transl Med. 2012;4(148):148ra16. 10.1126/scitranslmed.3004129 PMC3521604 22914622

[29] Unverdorben LV, Pirani A, Gontjes K, Moricz B, Holmes CL, Snitkin ES, *Klebsiella pneumoniae* evolution in the gut leads to spontaneous capsule loss and decreased virulence potential. mBio. 2025;16(5):e0236224. 10.1128/mbio.02362-24 PMC12077207 40162782

[30] Managò A, Becker KA, Carpinteiro A, Wilker B, Soddemann M, Seitz AP, *Pseudomonas aeruginosa* pyocyanin induces neutrophil death via mitochondrial reactive oxygen species and mitochondrial acid sphingomyelinase. Antioxid Redox Signal. 2015;22(13):1097–110. 25686490 10.1089/ars.2014.5979 PMC4403017

[31] Karges J. Reactive Oxygen Species Detection with Fluorescent Probes: Limitations and Recommendations beyond DCFH-DA. J Med Chem. 2026;69(3):1970–81. 41615811 10.1021/acs.jmedchem.5c03494

[32] Traore K, Trush MA, George M, Spannhake EW, Anderson W, Asseffa A. Signal transduction of phorbol 12-myristate 13-acetate (PMA)-induced growth inhibition of human monocytic leukemia THP-1 cells is reactive oxygen dependent. Leuk Res. 2005;29(8):863–79. 15978937 10.1016/j.leukres.2004.12.011

[33] Fontayne A, Dang PM, Gougerot-Pocidalo MA, El-Benna J. Phosphorylation of p47phox sites by PKC alpha, beta II, delta, and zeta: effect on binding to p22phox and on NADPH oxidase activation. Biochemistry. 2002;41(24):7743–50. 12056906 10.1021/bi011953s

[34] Guilliams M, Scott CL. Does niche competition determine the origin of tissue-resident macrophages? Nat Rev Immunol. 2017;17(7):451–60. 28461703 10.1038/nri.2017.42

[35] Thomas ST, Wierenga KA, Pestka JJ, Olive AJ. Fetal Liver-Derived Alveolar-like Macrophages: A Self-Replicating Ex Vivo Model of Alveolar Macrophages for Functional Genetic Studies. Immunohorizons. 2022;6(2):156–69. 35193942 10.4049/immunohorizons.2200011 PMC10217771

[36] Holmes CL, Dailey KG, Hullahalli K, Wilcox AE, Mason S, Moricz BS, Patterns of Klebsiella pneumoniae bacteremic dissemination from the lung. Nat Commun. 2025;16(1):785. 39824859 10.1038/s41467-025-56095-3 PMC11742683

[37] Holmes CL, Wilcox AE, Forsyth V, Smith SN, Moricz BS, Unverdorben LV, Klebsiella pneumoniae causes bacteremia using factors that mediate tissue-specific fitness and resistance to oxidative stress. PLoS Pathog. 2023;19(7):e1011233. 10.1371/journal.ppat.1011233 PMC10381055 37463183

[38] Nguyen GT, Shaban L, Mack M, Swanson KD, Bunnell SC, Sykes DB, SKAP2 is required for defense against *K. pneumoniae* infection and neutrophil respiratory burst. Elife. 2020;9. 10.7554/eLife.56656 PMC7250567 32352382

[39] Tsai WC, Strieter RM, Zisman DA, Wilkowski JM, Bucknell KA, Chen GH, Nitric oxide is required for effective innate immunity against *Klebsiella pneumoniae*. Infect Immun. 1997;65(5):1870–5. 9125574 10.1128/iai.65.5.1870-1875.1997 PMC175233

[40] Lu G, Zhang R, Geng S, Peng L, Jayaraman P, Chen C, Myeloid cell-derived inducible nitric oxide synthase suppresses M1 macrophage polarization. Nat Commun. 2015;6:6676. 25813085 10.1038/ncomms7676 PMC4389243

[41] Nguyen HK, Duke MM, Grayton QE, Broberg CA, Schoenfisch MH. Impact of nitric oxide donors on capsule, biofilm and resistance profiles of Klebsiella pneumoniae. Int J Antimicrob Agents. 2024;64(5):107339. 10.1016/j.ijantimicag.2024.107339 PMC11540743 39304122

[42] Paczosa MK, Silver RJ, McCabe AL, Tai AK, McLeish CH, Lazinski DW, Transposon Mutagenesis Screen of Klebsiella pneumoniae Identifies Multiple Genes Important for Resisting Antimicrobial Activities of Neutrophils in Mice. Infect Immun. 2020;88(4). 10.1128/IAI.00034-20 PMC7093148 31988174

[43] Wyres KL, Lam MMC, Holt KE. Population genomics of *Klebsiella pneumoniae*. Nat Rev Microbiol. 2020(6):344–59. 32055025 10.1038/s41579-019-0315-1

[44] Paczosa MK, Mecsas J. *Klebsiella pneumoniae*: Going on the Offense with a Strong Defense. Microbiol Mol Biol Rev. 2016;80(3):629–61. 27307579 10.1128/MMBR.00078-15 PMC4981674

[45] Xiong H, Keith JW, Samilo DW, Carter RA, Leiner IM, Pamer EG. Innate Lymphocyte/Ly6C(hi) Monocyte Crosstalk Promotes Klebsiella pneumoniae Clearance. Cell. 2016;165(3):679–89. 27040495 10.1016/j.cell.2016.03.017 PMC4842125

[46] Broberg CA, Wu W, Cavalcoli JD, Miller VL, Bachman MA. Complete Genome Sequence of Klebsiella pneumoniae Strain ATCC 43816 KPPR1, a Rifampin-Resistant Mutant Commonly Used in Animal, Genetic, and Molecular Biology Studies. Genome Announc. 2014;2(5). 10.1128/genomeA.00924-14 PMC4175196 25291761

[47] Rao K, Patel A, Sun Y, Vornhagen J, Motyka J, Collingwood A, Risk Factors for Klebsiella Infections among Hospitalized Patients with Preexisting Colonization. mSphere. 2021;6(3):e0013221. 10.1128/mSphere.00132-21 PMC8265626 34160237

[48] Kiritsy MC, Ankley LM, Trombley J, Huizinga GP, Lord AE, Orning P, A genetic screen in macrophages identifies new regulators of IFNγ-inducible MHCII that contribute to T cell activation. Elife. 2021;10. 10.7554/eLife.65110 PMC8598162 34747695

[49] Gilliland HN, Beckman OK, Olive AJ. A Genome-Wide Screen in Macrophages Defines Host Genes Regulating the Uptake of Mycobacterium abscessus. mSphere. 2023;8(2):e0066322. 10.1128/msphere.00663-22 PMC10117111 36794958

[50] Fejer G, Wegner MD, Györy I, Cohen I, Engelhard P, Voronov E, Nontransformed, GM-CSF-dependent macrophage lines are a unique model to study tissue macrophage functions. Proc Natl Acad Sci U S A. 2013;110(24):E2191–8. 23708119 10.1073/pnas.1302877110 PMC3683787

