# Supplementary material for: Effective macrophage clearance of Klebsiella pneumoniae requires the inducible nitric oxide synthase iNOS and is independent of reactive oxygen species generated by NADPH oxidase
Source: bioRxiv. 2026 May 18:2026.05.14.724925. Preprint. [Version 1] doi: 10.64898/2026.05.14.724925 (PMC13228317; doi:10.64898/2026.05.14.724925)
Supplement: Supplement 1 [file NIHPP2026.05.14.724925v1-supplement-1.pdf]

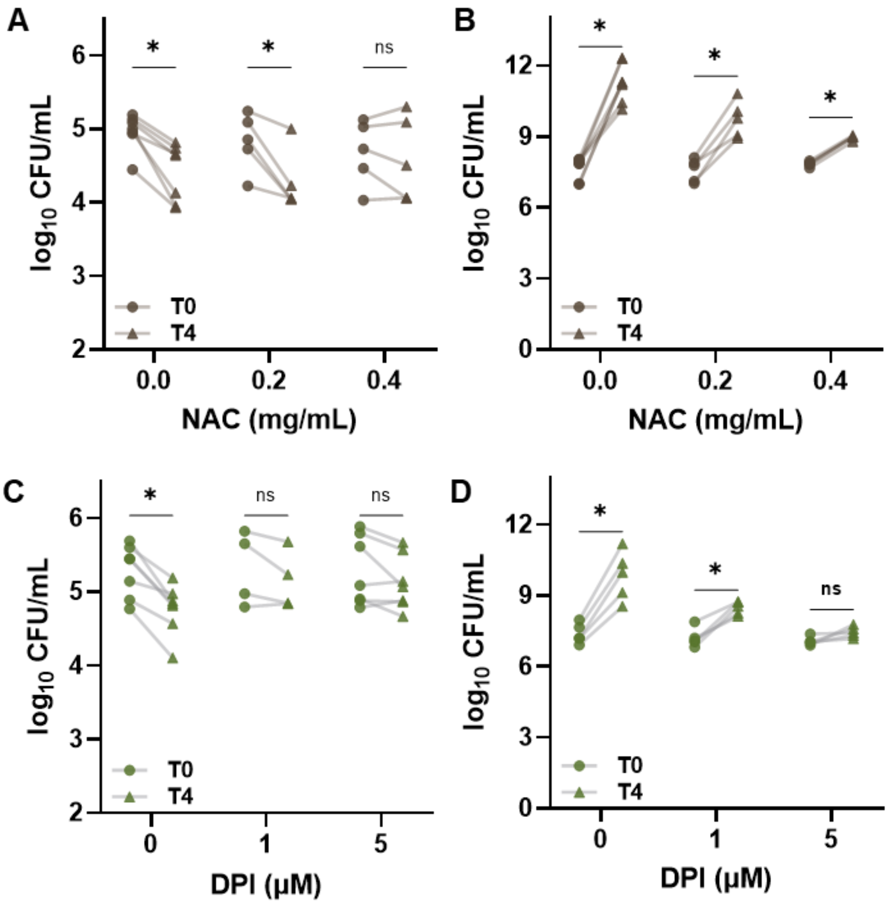

Supplemental Figure 1. Chemical inhibition of reactive species increases intracellular *K. pneumoniae* survival. Chemical inhibitors of reactive species were assessed for their ability to influence macrophage killing of intracellular *K. pneumoniae* and affect overall bacterial viability. (A-B) The antioxidant N-acetyl-cysteine (NAC) was used to broadly inhibit reactive species at a concentration of 0.2mg/mL and 0.4mg/mL. (C-D) Diphenyleneiodonium (DPI) was used to inhibit NOX2 and iNOS activity at a concentration of 1μM and 5μM. In (A) and (C), immortalized bone marrow derived macrophages were either left untreated or treated with each inhibitor at the indicated concentration. Cells were infected with KPPR1 for one hour prior to the removal of extracellular bacteria with gentamicin treatment. Abundance of intracellular bacteria was assessed either immediately (T0) or after four hours (T4). *$p<0.05$ by paired *t*-test comparing log CFU/mL at T0 and T4 for each treatment. In (B)

bioRxiv preprint doi: https://doi.org/10.64898/2026.05.14.724925; this version posted May 18, 2026. The copyright holder for this preprint (which was not certified by peer review) is the author/funder, who has granted bioRxiv a license to display the preprint in perpetuity. It is made available under a CC-BY 4.0 International license.

and (D), the influence of each inhibitor on KPPR1 viability was assessed by incubating the bacteria with the inhibitors in culture media alone without macrophages. Bacterial abundance initially (T0) and after four hours (T4) was assessed by quantitative culture. *$p<0.05$ by paired *t*-test comparing log CFU/mL at T0 and T4 for each treatment. In all, n=5-6 independent trials with symbols representing independent experiments.

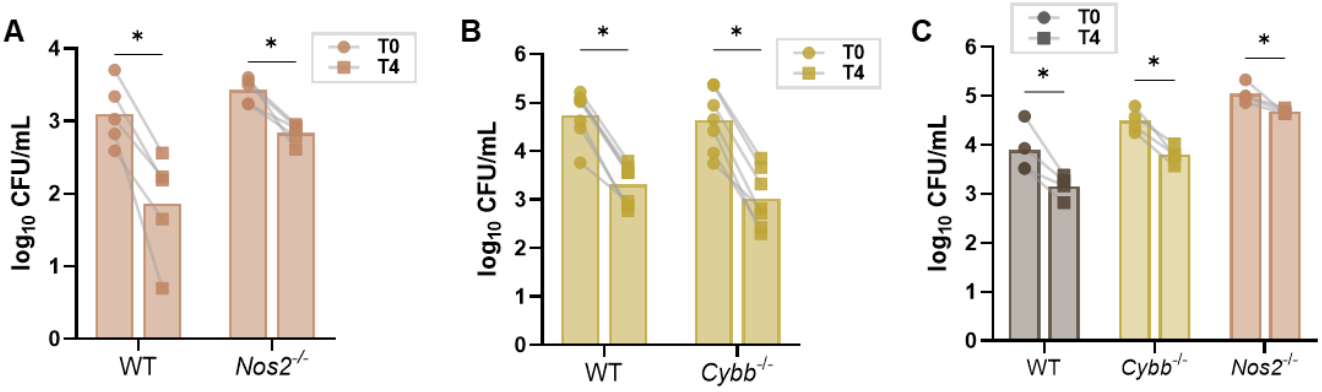

Supplemental Figure 2. KPPR1 abundance recovered from macrophages. Wild-type, (A) *Nos2*$^{-/-}$, or (B) *Cybb*$^{-/-}$ primary bone marrow-derived macrophages were assessed for the ability to kill intracellular KPPR1. (C) The results for WT, *Nos2*$^{-/-}$, and *Cybb*$^{-/-}$ cells were validated using immortalized bone marrow-derived macrophages. Cells were infected with KPPR1 for one hour prior to the removal of extracellular bacteria with gentamicin treatment. Abundance of intracellular bacteria was assessed either immediately (T0) or after four hours (T4). For all, *$p<0.05$ by paired *t*-test; n=4-6 trials with symbols representing independent experiments and the tops of bars representing the mean value.

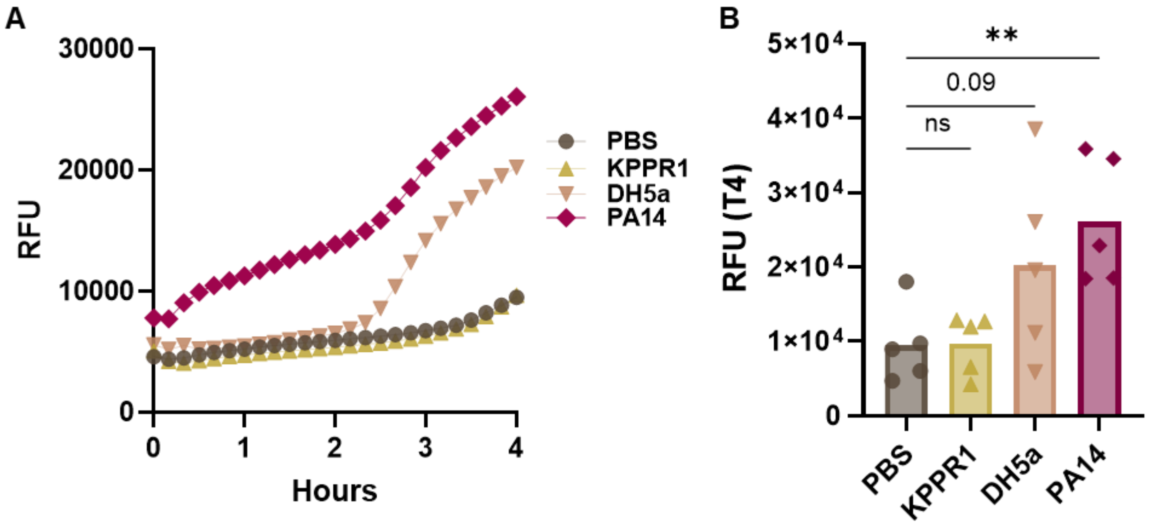

Supplemental Figure 3. Macrophage ROS in response to infection from other Gram-negative species. Immortalized bone marrow derived macrophages were left untreated (PBS), or infected with *K. pneumoniae* (KPPR1), *Escherichia coli* (DH5α), or *Pseudomonas aeruginosa* (PA14) for 1 hour. Cells were then stained for ROS using DCFDA and fluorescence was measured every 10 minutes for 4 hours. (A) The mean DCFDA relative fluorescence (RFU) is displayed for each species. (B) The RFU at T4 for the data displayed in (A). For (B) **$p<0.01$ by paired one-way ANOVA comparing each group to the untreated control (PBS). The top of each bar represents the mean RFU group value, n=5 and symbols represent independent trials.
